# Genetic polymorphism of prolactin and nitric oxide synthase in Holstein cattle

**DOI:** 10.14202/vetworld.2023.161-167

**Published:** 2023-01-27

**Authors:** Indira Saltanovna Beishova, Alena Valentinovna Belaya, Yusupzhan Artykovich Yuldashbayev, Gulzhagan Dzhambulovna Chuzhebayeva, Vadim Alexandrovich Ulyanov, Tatyana Vladimirovna Ulyanova, Alexandr Mikhailovich Kovalchuk, Ulbolsyn Zhangaziyevna Kuzhebayeva, Aidar Myrzahmetuly Namet

**Affiliations:** 1 Non-profit JSC “Zhangir Khan West Kazakhstan Agrarian Technical University”, Uralsk, Republic of Kazakhstan; 2 Belarusian State Pedagogical University named after Maxim Tank, Minsk, Republic of Belarus; 3 Russian State Agrarian University - Moscow Timiryazev Agricultural Academy, Moscow, Russian Federation; 4 Non-profit JSC “A. Baitursynov Kostanay Regional University”, Kostanay, Republic of Kazakhstan; 5 MVA Group “Scientific-Research Production Center” Ltd., Almaty, Republic of Kazakhstan

**Keywords:** bovine leukemia, cattle, Holstein breed, mastitis, polymerase chain reaction-restriction fragment length polymorphism, polymorphism

## Abstract

**Background and Aim:**

Bacterial and viral infections affect the welfare of animals and lead to large economic losses in dairy cattle breeding due to decreased productive indicators and increased culling rates. In modern dairy farming, farmers are looking for effective solutions to prevent and minimize infectious disease risks. To this end, the most relevant study field is the search for gene sites that impact production and health. This study aimed to determine the nature of the distribution of the relative frequencies of alleles and genotypes of polymorphic prolactin (*PRL*) and nitric oxide synthase (*NOS2*) in Holstein cows and identify the relationship of these genes with resistance to mastitis and bovine leukemia.

**Materials and Methods:**

For this study, we chose cows because infectious diseases affect the amount of lactation and milk quality. Holstein cattle with mastitis and bovine leukemia were selected. Animal genotypes were determined by restriction fragment length polymorphism (RFLP) of polymerase chain reaction (PCR) products. The results were analyzed using a nonparametric statistical method using Microsoft Excel 2010 and Statistica 6.0.

**Results:**

In healthy animals, 94 genotypes were identified for both genes under study. For *bPRL*, *bPRL*-RsaI^AA^ (72) was the most common genotype and *bPRL*-RsaI^BB^ (4) the least; for *NOS2*, *bNOS2***-**HinfI^AB^ (47) was the most common genotype and *bNOS2***-**HinfI^AA^ the least (21). In animals with leukemia, 34 genotypes were identified. For *PRL*, *bPRL*-RsaI^AA^ (25) was the most common genotype and *bPRL*-RsaI^BB^ (2) the least; for *NOS2*, *bNOS2***-**HinfI^BB^ (17) was the most common genotype and *bNOS2***-**HinfI^AA^ (3) the least. In animals with mastitis, 67 genotypes were identified. For *PRL*, *bPRL*-RsaI^AA^ (43) was the most common genotype and *bPRL*-RsaI^BB^ (6) the least; for *NOS2*, *bNOS2***-**HinfI^BB^ (31) was the most common genotype and *bNOS2*-HinfI^AA^ (7) the least. The distribution of genotypes of polymorphic *bPRL* and *bNOS2* generally coincides, and *bPRL*-RsaI^BB^ is the most common genotype. In groups of sick animals, the number of *bNOS2***-**HinfI^AA^ homozygotes was lower than that of the control group. In particular, the proportion of animals with the *bNOS2***-**HinfI^AA^ genotype with bovine leukemia was 8.7% and with mastitis was 10.3% compared with 22.4% in healthy animals. These data support the possible association of the *bNOS2***-**HinfI^AA^ genotype with resistance to infection. The frequency of the *bPRL*-RsaI^B^ allele was higher in groups of sick animals. This allele is associated with increased milk productivity, suggesting that highly productive animals are less resistant to the incidence of viral bovine leukemia and mastitis of bacterial etiology.

**Conclusion:**

DNA amplification of Holstein cattle for the polymorphic regions of *PRL* and *NOS2* using the PCR-RFLP method revealed a possible connection between the distribution of relative allele frequencies of *bPRL* and *bNOS2* and resistance to viral and bacterial infections. Thus, in groups of sick animals, the frequency of *bPRL-*RsaI^BB^, associated with increased milk production compared with the theoretically calculated equilibrium value was higher and the number of homozygotes *bNOS2***-**HinfI^AA^ was lower than in the control group. In conclusion, animals with increased milk production were more prone to diseases, such as mastitis and bovine leukemia.

## Introduction

In Kazakhstan, Holstein is one of the predominant breeds of dairy cattle. The popularity of this breed is due to its high productivity [1]. However, a significant increase in milk production strains the animal’s body, leading to decreased resistance to adverse environmental conditions, increased occurrence of infectious diseases, and decreased productivity [2].

An important role in increasing the productivity of the dairy industry is played not only by modern technologies of cattle breeding and selection but also by the use of modern methods of combating infectious diseases [3]. In Kazakhstan, mastitis [4] and bovine leukemia [5, 6] are the most common infectious bacterial and viral diseases in cattle. Economic damage from animal diseases is caused by a decrease in the quality and quantity of productivity indicators [7, 8], premature culling of highly productive individuals [9, 10], disturbance of breeding work, loss of valuable breed gene pool [11], and extra costs incurred for meeting the requirements of veterinary regulations [12, 13].

Therefore, in addition to the use of marker-associated breeding measures for increasing the productivity of farm animals, the use of genetic markers of resistance to bacterial and viral infections can increase profitability by reducing treatment costs and losses from mortality, abortibility, and culling of sick animals. The study and identification of genetic markers of resistance to common infections such as mastitis and bovine leukemia and their involvement in breeding programs to improve disease resistance can significantly contribute to the development of breeding and veterinary medicine.

Prolactin (PRL), a regulator of lactation in mammals, including cattle, has significant potential as a candidate gene for increased milk productivity. Prolactin, like growth hormones, belongs to the family of protein hormones that are involved in the initiation and maintenance of lactation in mammals [14]. In cattle, *PRL* is located on chromosome 23 and contains five exons and four introns [15]. Its transcription is regulated by two independent promoter regions: The proximal one controls pituitary-specific expression [16], whereas the distal one is responsible for extrahypophysial gene expression [17, 18]. Prolactin is produced by lactotrophs, cells of the anterior pituitary gland [19]. Prolactin has been reported to be synthesized in various tissues, including endothelial cells, neurons, and breast cells [20]. Prolactin secretion is regulated by pituitary cells (paracrine regulation), as well as by several intracellular factors secreted by lactotrophic cells (autocrine regulation).

The cytokine system is a universal, polymorphic, regulatory network of mediators that control proliferation, differentiation, apoptosis, and the functional activity of cellular elements in the hematopoietic, immune, and homeostatic systems [21]. One of the main aspects of the functioning of the cytokine network is the presence of several polymorphic sites in the promoter regions of these genes. The differences in the transcription sites of the promoter regions have a great influence on the level of mRNA synthesis and the production of regulatory proteins. Individual differences in these gene regions affect the course of the main systems of vital activity of the body and its state of health [22].

Cytokines are mainly formed in immune cells and are factors of intercellular interaction that enable cells to contact each other at a distance and perform their functions. Pro-inflammatory cytokines (chemokines) play an important role in the study of the molecular mechanisms of the occurrence of pathological processes. In turn, they interact with the mediator of apoptosis, with the regulator of the innate immunity system, short-lived nitric oxide (NO). Nitric oxide mediates the cytotoxic effect of the immune cells or can stop the growth of pathogenic microorganisms [23, 24]. In cattle, NO synthase (*NOS2*) is located on chromosome 19 and contains 26 exons and 25 introns [25]. Inducible NO synthase encoded by *NOS2* is a cytoplasmic protein and is absent in resting cells, but is rapidly produced in response to stimuli, such as infections and cytokines [26].

These data suggest that some combinations of polymorphic variants of *PRL* involved in the control of lactation and *NOS2* associated with infection resistance can lead to increased resistance in animals against the background of high lactation intensity.

This study aimed to determine the nature of the distribution of relative frequencies of alleles and genotypes of polymorphic *PRL* and *NOS2* in Holstein cows and to identify the relationship of polymorphisms with resistance to viral and bacterial infections.

## Materials and Methods

### Ethical approval

All procedures performed in this study followed ethical standards. The study was approved by the National Scientific Council of the National Center for State Scientific and Technical Expertise of the Science Committee of the Ministry of Education and Science of the Republic of Kazakhstan on the priority project “Sustainable development of the agro-industrial complex and the safety of agricultural products.”

### Study period and location

The study was conducted from November 2021 to July 2022 in the Laboratory of Biotechnology and Diagnostics of Infectious Diseases of the Non-profit JSC “Zhangir Khan West Kazakhstan Agrarian Technical University.”

### Animals and sampling

Animals with established diagnoses of mastitis and bovine leukemia were selected from five farms in the Kostanay and West Kazakhstan regions of the Republic of Kazakhstan. Samples of biomaterials from healthy animals were selected from the same farms to form a control sample. Holstein cows born in 2018–2019 (up to the third lactation) were divided into three groups. The first group included 34 heads diagnosed with bovine leukemia, the second included 67 heads with a diagnosis of mastitis, and the third (the control group of healthy animals) included 94 heads.

### Study design

We analyzed the correspondence of genotype distribution for the studied polymorphic genes to the theoretically expected one, according to the Hardy–Weinberg principle. In the first stage, we evaluated milk productivity in the healthy control group with different genotypes according to the *bPRL* polymorphism. Then, to analyze the association between the allelic variants of *bNOS2* and *bPRL* and resistance to mastitis and leukemia, an assessment was made of the correspondence of the actual frequencies of genotypes theoretically expected by the Hardy–Weinberg principle, as well as by comparing the frequency distribution of alleles of the studied genes in groups of sick animals and healthy controls and assessing the reliability of the observed differences. For this, the relative frequencies of allelic variants in each group were calculated.

For cattle genotyping, we used animal hair follicles. This biological material was preferred due to the simple selection procedure and because no special conditions are required for transportation. The subclinical form of mastitis in cows was determined by examining the secretion of the mammary gland using mastitis tests with mastoprim using the Somatos-Mini device (Sibagropribor LLC, Russia), as well as bacteriological examination of the samples for the presence of the main pathogens of mastitis. Milk sampling was performed per GOST 13928-84 [27], using the MM-04B zootechnical control device for milk (Gomelagrokomplekt JSC, Belarus). Milk yield in animals was determined using individual counters. Fat and protein contents were determined using the Lactan 1-4M device (Sibagropribor LLC MIC, Russia). Genomic DNA was extracted from animal samples using the DNA-Extran-2 set of reagents (Syntol LLC, Russia). Genotype detection by selected polymorphisms was performed using the polymerase chain reaction (PCR)-restriction fragment length polymorphism (RFLP) method. The primer sequences and PCR conditions for the analysis of each polymorphism are presented in Table-1 [28, 29].

**Table-1:** Individual characteristics of PCR conditions for the studied polymorphic loci of *bPRL* and *bNOS2* genes.

Polymorphism	Amplification conditions	Primer sequences	Reference
*bPRL*-RsaI	95°C: 5 min; (95°C: 30 s; 63°C: 30 s; 72°C: 30 s) × 35° cycles; 72°C: 10 min	RsaI-F: 5′-gctccagaagtcgttgttttc-3′ RsaI-R: 5′-cgagcttatgagcttgattctt-3′	[28]
*bNOS2***-**HinfI	94°C: 4 min; (94°C: 10 s; 58°C: 10 s; 72°C: 10 s) × 35° cycles; 72°C: 5 min	HinfI-F: 5’- agaggccagagaggaagaag-3’ HinfI-R: 5’- ggaccctaacctcgaagactg-3’	[29]

PCR=Polymerase chain reaction

### Identification of the nucleotide sequence polymorphism of *bPRL*

**Figure-1:**
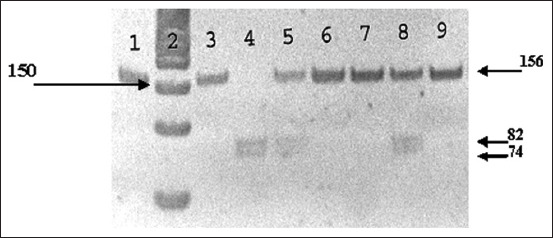
Electrophoregram of DNA typing of polymorphism *bPRL*-RsaI.

Identification of the nucleotide sequence polymorphism of *bPRL* in exon 3 was performed using the RsaI restrictase (Figure-1). The *bPRL*-RsaI polymorphism is due to the silent A→G transversion corresponding to position 103 of the amino acid sequence. The GT↓AC sequence is the recognition site for RsaI. The restrictase cuts the amplicon containing the nucleotide A. This allele is designated as *bPRL*-RsaI^B^. In the presence of the G nucleotide, the restriction site disappears, and this allele is designated as *bPRL*-RsaI^A^ [28]. The length of the amplified fragment was 156 bp. The length of the fragments after restriction was 82 and 74 bp. On the electrophoregram, variants of bands of lengths that are characteristic of genotypes can be visualized: One band of 156 bp (*bPRL*-RsaI^AA^); two bands of 82 and 74 bp (*bPRL*-RsaI^BB^); and three bands of 156, 82, and 74 bp (*bPRL*-RsaI^AB^).

### Identification of the nucleotide sequence polymorphism of *bNOS2*

**Figure-2:**
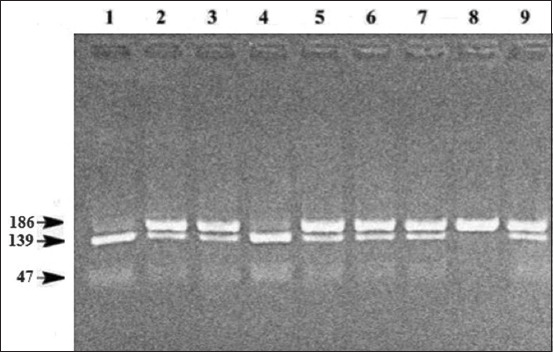
Electrophoregram of DNA typing of polymorphism *bNOS2*-HinfI.

The nucleotide sequence polymorphism of *bNOS2* in intron 6 was identified using the HinfI restrictase (rs136843322 polymorphism) (Figure-2). The *bNOS2*-HinfI polymorphism was caused by a G→A substitution (g.19412724G>A). The G↓ANTC sequence was the recognition site for HinfI. The length of the amplified fragment was 186 bp. The length of the fragments after restriction was 139 and 47 bp. On the electrophoregram, variants of bands of lengths that are characteristic of genotypes can be visualized: One band of 186 bp (*bNOS2*-HinfI^BB^); two bands of 47 and 139 bp (*bNOS2*-HinfI^AA^); and three bands of 186, 139, and 47 bp (*bNOS2*-HinfI^AB^).

### Statistical analysis

The genotype of each animal for all analyzed genes was documented and entered into a common database. For groups of animals with different genotypes, the average values of milk yield, fat content, and protein content were calculated for 305 days of lactation. Statistical evaluation of differences between groups with three possible genotypes was performed by the method of rank analysis of variations for three or more independent groups using the Kruskal–Wallis test. The differences were considered statistically significant at a significance level of p < 0.05. The significance of the observed deviations was assessed using the c^2^ criterion. Statistical data were processed using Microsoft Excel 2010 (Microsoft Corporation, USA) and STATISTICA 6.0 (StatSoft, Inc., USA).

## Results

The milk productivity of healthy cows with different genotypes of *bPRL* was evaluated to determine and characterize the preferred genotype. Table-2 shows the p values for the comparative Kruskal–Wallis test and indicators of milk productivity for healthy cows with different genotypes of *bPRL*-RsaI polymorphism.

**Table-2:** Characteristics of milk productivity in healthy cows with different genotypes of *bPRL*-RsaI polymorphism (m ± σ).

Genotype	n	Milk yield for 305 days of lactation, kg	Amount of milk fat for 305 days of lactation, kg	Amount of milk protein for 305 days of lactation, kg
*bPRL*-RsaI^BB^	4	7,310 ± 169	226 ± 12	235 ± 11
*bPRL*-RsaI^AB^	18	6,807 ± 142	215 ± 9	224 ± 8
*bPRL*-RsaI^AA^	72	6,562 ± 114	209 ± 5	213 ± 5
p*		0.053	0.735	0.074

* The p-value for the Kruskal-Wallis comparative test

Holstein cows with the *bPRL*-RsaI^BB^ genotype tend to have increased milk yield (7,310 ± 169 kg) compared with those with the *bPRL*-RsaI^AB^ genotype (6,807 ± 142 kg) (Table-2). However, this observation does not allow us to consider this genotype as the preferred one for this feature because the p-value of the Kruskal–Wallis test statistics exceeds 0.05. Table-3 shows the characteristics of samples of sick and healthy animals according to the nature of the distribution of genotypes of the studied genes.

**Table-3:** Distribution of genotype frequencies of *bPRL* and *bNOS2* polymorphic genes in groups of healthy and sick animals.

Genotype	Healthy (n = 94)			Bovine leukemia (n = 34)			Mastitis (n = 67)		
		
	n_o_	n_e_	χ^2^	n_o_	n_e_	χ^2^	n_o_	n_e_	χ^2^
*bPRL*-RsaI^BB^	4	2	3.63	2	1	1.97	6	3	3.45
*bPRL*-RsaI^AB^	18	22		7	9		18	23	
*bPRL*-RsaI^AA^	72	70		25	24		43	41	
*bNOS2*-HinfI^AA^	21	21	0.00	3	4	0.33	7	9	0.30
*bNOS2*-HinfI^AB^	47	47		14	16		29	31	
*bNOS2*-HinfI^BB^	26	26		17	14		31	27	

The χ^2^ value for a significance level of 0.05 is 3.84. *The χ^2^ values were calculated with the Yates correction

The nature of the distribution of genotypes of polymorphic *bPRL* and *bNOS2* generally coincides in animals with bovine leukemia and mastitis and in healthy animals (Table-3). In particular, for the *bPRL*-RsaI polymorphism, the *bPRL*-RsaI^BB^ genotype is the most common in all three groups. In all groups, the c^2^ value is quite high; however, the nature of the distribution corresponds to the equilibrium.

Notably, in cows diagnosed with bovine leukemia and mastitis, the observed number of animals with *bPRL*-RsaI^BB^ associated with increased milk productivity exceeded the theoretically calculated equilibrium value. This may indicate that animals with increased milk production are more susceptible to both viral bovine leukemia and bacterial mastitis. Regarding *bNOS2***-**HinfI, in the group of healthy animals, the expected number of genotypes coincided. In sick animals, a slight decrease in the observed *bNOS2***-**HinfI^AA^ genotypes was observed compared with the theoretically expected one according to the Hardy–Weinberg principle.

**Figure-3:**
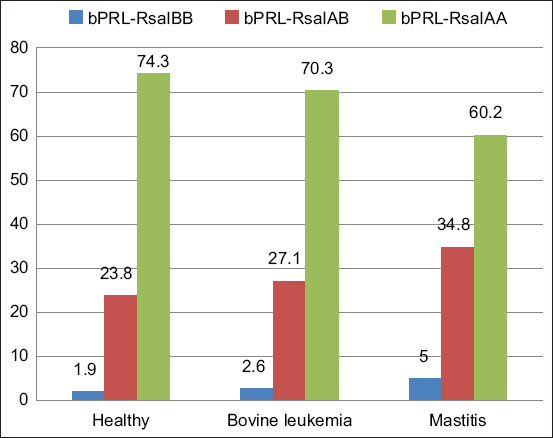
Relative frequencies of *bPRL*-RsaI polymorphism genotypes in the studied animal groups.

**Figure-4:**
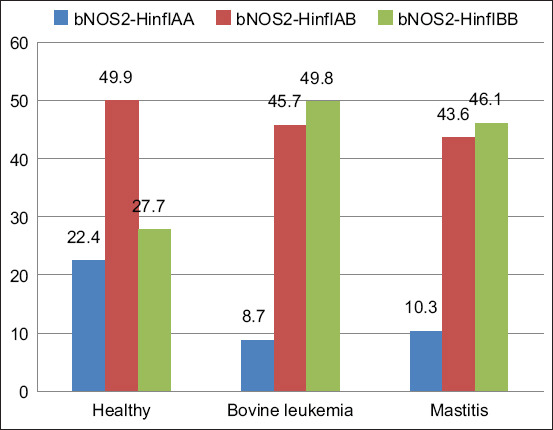
Relative frequencies of *bNOS2***-**HinfI polymorphism genotypes in the studied animal groups.

In cows with bovine leukemia and mastitis, the number of *bNOS2***-**HinfI^AA^ homozygotes was lower than that in the control group (Figures-3 and 4). In particular, 8.7% and 10.3% of animals with *bNOS2***-**HinfI^AA^ had bovine leukemia and mastitis, respectively, compared with 22.4% in healthy animals. These data support the possible association of *bNOS2***-**HinfI^AA^ with resistance to viral and bacterial infections in cattle. The characteristic of the distribution of relative frequencies of alleles of polymorphic *bPRL* and *bNOS2* in groups of sick and healthy Holstein cows is shown in Table-4.

**Table-4:** Distribution of relative frequencies of alleles of the studied *bPRL* and *bNOS2* genes.

Polymorphism	Allele	Healthy (n = 94)	Bovine leukemia (n = 34)	Mastitis (n = 67)
*bPRL*-RsaI	*bPRL*-RsaI^B^	0.138 ± 0.02	0.162 ± 0.01	0.224 ± 0.02
	*bPRL*-RsaI^A^	0.862 ± 0.02	0.838 ± 0.01	0.776 ± 0.02
*bNOS2***-**HinfI	*bNOS2***-**HinfI^A^	0.473 ± 0.01	0.294 ± 0.02	0.321 ± 0.04
	*bNOS2***-**HinfI^B^	0.527 ± 0.01	0.706 ± 0.02	0.679 ± 0.04

The frequency of the *bPRL*-RsaI^B^ allele associated with increased milk productivity is higher in groups of sick animals, which suggests that highly productive animals are less resistant to viral bovine leukemia and mastitis of bacterial etiology (Table-4).

## Discussion

Increasing milk yield is of paramount importance in dairy cattle breeding. One of the main features in the selection of animals to improve milk yield is the assessment of genotypic value. In this study, we performed genotyping by polymorphic genes from the Holstein breed of cattle. This breed is the most common dairy cattle in the world and has been recognized for its high milk productivity. However, if animals with high milk yields are not properly maintained, they can be more susceptible to infectious diseases, such as mastitis and bovine leukemia.

When determining the nature of dairy productivity of healthy cows with different genotypes of the *bPRL*-RsaI polymorphism, we found that Holstein cows with *bPRL*-RsaI^BB^ tended to have increased milk yield compared with the group with *bPRL*-RsaI^AB^, where the *P* value of the Kruskal–Wallis test statistics exceeded 0.05. On this basis, this was not the preferred genotype. In the study by Ozdemir *et al*. [30], 186 heads of Holstein cattle for *PRL* had the following frequency of the genotype: RsaI^−/−^: 0.26, RsaI^+/+^: 0.22, and RsaI^−/+^: 0.52. The authors determined that the relationship between the polymorphism of the PRL/RsaI gene and the studied milk yield indicators was not significant, which correlates with our results. According to Oğuzkan and Bozkurt [31], high milk productivity in Holstein cows was associated with *bPRL*-RsaI^AA^. In a study conducted by Gilmanov *et al*. [32], the frequency of occurrence of alleles of the PRL/A gene was 0.90, for PRL/B was 0.10, and for the individual genotypes of this gene was the following: PRL/AA: 79.7% and PRL/AB: 20.3%, when using the PCR-RFLP method. The authors noted that the PRL/BB genotype was not detected in the studied population of black-and-white Holstein cows, which influenced the emergence of complex genotypes.

The incidence of mastitis in animals is highest during calving, which is associated with increased PRL production. In the studies of Salgado-Lora *et al*. [33] and Lara- Zárate *et al*. [34], PRL was suggested to play a role in the development of mastitis. The authors found that PRL promoted an inflammatory reaction in the epithelial cells of the mammary gland of cattle through the activity of nuclear factor kappa B. We determined that in groups of cows diagnosed with mastitis, number of animals with *bPRL*-RsaI^BB^ associated with increased milk productivity exceeded the theoretically calculated equilibrium value, which may indicate that animals with increased milk productivity are more susceptible to mastitis.

In this study, when determining the *bNOS2***-**HinfI polymorphism in healthy animals, the expected number of genotypes coincided with the expected one. However, in sick animals, a slight decrease was noted in the observed *bNOS2***-**HinfI^AA^ genotypes compared with those theoretically expected by the Hardy–Weinberg principle. Chichinina, when studying a sample of 54 heads of seropositive (AGID+) Holstein cattle, obtained the following relative frequencies of genotypes of the polymorphic variant of *bNOS2*: AA genotype: 59%, AB genotype: 13%, and BB genotype: 28%. In the study with a sample of 50 heads of seronegative (AGID−) Holstein cattle, the following relative frequencies of genotypes of the polymorphic variant of *bNOS2* were obtained: AA genotype: 63%, AB genotype: 22%, and BB genotype: 15% [35]. Gilmanov, when studying a sample of 60 heads of mixed and purebred Holstein cattle, obtained the following relative frequencies of genotypes of the polymorphic variant of *bNOS2*: AA genotype: 23.3%, AB genotype: 58.3%, and BB genotype: 18.4%. In the study with a sample of eight heads of beef cattle represented by the Hereford, Aubrac, and Limousin breeds, the following relative frequencies of genotypes of the polymorphic *bNOS2* were obtained: AA genotype: 50%, AB genotype: 50%, and BB genotype: 0% [36].

## Conclusion

The findings of this study revealed that Holstein cows with the *bPRL-RsaI*^BB^ genotype tended to increase milk yield (7,310 ± 169 kg) compared with the group with the *bPRL-RsaI*^AB^ genotype (6,807 ± 142 kg). In animals diagnosed with bovine leukemia and mastitis, an excess of the relative frequency of *bPRL-RsaI*^BB^ was associated with increased milk productivity compared with the theoretically calculated equilibrium value. This may indicate that animals with increased milk production are more susceptible to both viral bovine leukemia and bacterial mastitis.

In cows with bovine leukemia and mastitis, the number of *bNOS2***-**HinfI^AA^ homozygotes was lower than that in the control group. In particular, the proportion of animals with *bNOS2***-**HinfI^AA^ and bovine leukemia and mastitis was 8.7% and 10.3%, respectively, compared with 22.4% in healthy animals. These data support the possible association of the *bNOS2***-**HinfI^AA^ genotype with resistance to viral and bacterial infection in cattle.

## Authors’ Contributions

ISB: Developed the concept and design of this study and participated in the writing of the manuscript. AVB: Developed the concept and design of the study, performed the analysis and interpretation of the data, and participated in the writing of the manuscript. YAY: Developed the concept and design of this study and participated in the writing of the manuscript. GDC: Performed the analysis and interpretation of the data and participated in the writing of the manuscript. VAU: Established the cattle genotypes and carried out statistical processing. TVU: Established the cattle genotypes, carried out statistical processing, and participated in the writing of the manuscript. AMK: Carried out the selection of biological material and performed statistical processing. UZK: Performed the analysis and interpretation of the data and participated in the writing of the manuscript. AMN: Carried out statistical analysis. All authors have read and approved the final manuscript.
